# Association between the regional variation in premature mortality and immigration in Ontario, Canada

**DOI:** 10.17269/s41997-020-00330-5

**Published:** 2020-05-27

**Authors:** Laura C. Rosella, Kathy Kornas, Tristan Watson, Emmalin Buajitti, Catherine Bornbaum, David Henry, Adalsteinn Brown

**Affiliations:** 1 Dalla Lana School of Public Health, Health Sciences Building, University of Toronto, 6th floor, 155 College Street, Toronto, Ontario M5T 3M7 Canada; 2 ICES, Toronto, ON Canada; 3 Public Health Ontario, Toronto, ON Canada; 4 Health & Rehabilitation Sciences, Faculty of Health Sciences, Western University, London, ON Canada

**Keywords:** Premature mortality, Regional variation, Immigrants, Healthy immigrant effect, Mortalité prématurée, variation régionale, immigrants, effet de l’immigrant en bonne santé

## Abstract

**Objectives:**

Health region differences in immigration patterns and premature mortality rates exist in Ontario, Canada. This study used linked population-based databases to describe the regional proportion of immigrants in the context of provincial health region variation in premature mortality.

**Methods:**

We analyzed all adult premature deaths in Ontario from 1992 to 2012 using linked population files, Canadian census, and death registry databases. Geographic boundaries were analyzed according to 14 health service regions, known as Local Health Integration Networks (LHINs). We assessed the role of immigrant status and regional proportion of immigrants in the context of these health region variations and assessed the contribution using sex-specific multilevel negative binomial models, accounting for age, individual- and area-level immigration, and area-level material deprivation.

**Results:**

We observed significant premature mortality variation among health service regions in Ontario between 1992 and 2012. Average annual rates ranged across LHINs from 3.03 to 6.40 per 1000 among males and 2.04 to 3.98 per 1000 among females. The median rate ratio (RR) decreased for men from 1.14 (95% CI 1.06, 1.19) to 1.07 (95% CI 1.00, 1.11) after adjusting for year, age, area-based material deprivation, and individual- and area-level immigration, and among females reduced from 1.13 (95% CI 1.05, 1.18) to 1.04 (95% CI 1.00, 1.05). These adjustments explained 84.1% and 94.4% of the LHIN-level variation in males and females respectively. Reduced premature mortality rates were associated with immigrants compared with those for long-term residents in the fully adjusted models for both males 0.43 (95% CI 0.42, 0.44) and females 0.45 (0.44, 0.46).

**Conclusion:**

The findings demonstrate that health region differences in premature mortality in Ontario are in part explained by individual-level effects associated with the health advantage of immigrants, as well as contextual area-level effects that are associated with regional differences in the immigrant population. These factors should be considered in addition to health system factors when looking at health region variation in premature deaths.

**Electronic supplementary material:**

The online version of this article (10.17269/s41997-020-00330-5) contains supplementary material, which is available to authorized users.

## Résumé

**Objectifs:**

En Ontario (Canada), les tendances de l’immigration et les taux de mortalité prématurée varient d’une région sanitaire à l’autre. Nous avons utilisé des bases de données représentatives couplées pour décrire la proportion régionale d’immigrants dans le contexte de la variation de la mortalité prématurée selon la région sanitaire.

**Méthode:**

Nous avons analysé tous les décès prématurés d’adultes survenus en Ontario entre 1992 et 2012 à l’aide des bases de données couplées de fichiers de population, du Recensement du Canada et du registre des décès. Les frontières géographiques ont été analysées en fonction des 14 régions de services de santé de la province : les réseaux locaux d’intégration des services de santé (RLISS). Nous avons évalué le rôle du statut d’immigrant et la proportion régionale d’immigrants dans le contexte de ces variations entre régions sanitaires, ainsi que la contribution du facteur de l’immigration à l’aide de modèles binomiaux négatifs multiniveaux spécifiques selon le sexe et tenant compte de l’âge, de l’immigration individuelle et régionale, et de la défavorisation matérielle régionale.

**Résultats:**

Nous avons observé une variation significative de la mortalité prématurée entre les régions sanitaires de l’Ontario entre 1992 et 2012. Les taux annuels moyens dans les RLISS s’échelonnaient de 3,03 à 6,40 pour 1 000 chez les hommes et de 2,04 à 3,98 pour 1 000 chez les femmes. Chez les hommes, le rapport de taux (RT) médian a diminué, passant de 1,14 (IC de 95 % 1,06, 1,19) à 1,07 (IC de 95 % 1,00, 1,11) après ajustements pour tenir compte de l’année, de l’âge, de la défavorisation matérielle régionale et de l’immigration individuelle et régionale; chez les femmes, le RT est passé de 1,13 (IC de 95 % 1,05, 1,18) à 1,04 (IC de 95 % 1,00, 1,05). Ces ajustements ont expliqué 84,1 % et 94,4 % de la variation entre les RLISS pour les hommes et les femmes, respectivement. Les taux de mortalité prématurée réduits étaient associés aux immigrants comparativement aux résidents de longue date dans le modèle entièrement ajusté, tant chez les hommes, à 0,43 (IC de 95 % 0,42, 0,44), que chez les femmes, à 0,45 (0,44, 0,46).

**Conclusion:**

Ces constatations montrent que les différences dans la mortalité prématurée entre les régions sanitaires de l’Ontario s’expliquent en partie par les effets individuels associés à la meilleure santé des immigrants, ainsi qu’aux effets régionaux contextuels associés aux différences régionales dans la population immigrante. Il faudrait tenir compte de ces facteurs, en plus des facteurs liés au système de santé, lorsqu’on étudie la variation des décès prématurés entre les régions sanitaires.

## Introduction

In the 20-year period spanning 1992 and 2012, Ontario, Canada’s most populous province, achieved substantial declines in all-cause mortality rates by 40% in men and 30% in women (Rosella et al. 2016). Despite the overall reductions in mortality, a geographic gradient in mortality exists in that the centrally located regions of Ontario generally have shown lower all-cause and premature mortality rates compared with those regions located in the eastern, northern, and western boundaries of the province (Buajitti et al. 2018). The spatial patterning of mortality across geographic regions has also been observed in other jurisdictions (Dwyer-Lindgren et al. 2016).

The socio-demographic compositions of the population living within a region, which coincide with how risk factors are distributed, are important factors for mortality trends (Omran 2005). Ontario exhibits a unique population structure with an estimated 29.1% of the total population comprised of immigrants as of 2016 (Statistics Canada 2017). The majority of immigrants to Ontario settle in major urban cities located centrally in the Greater Toronto census metropolitan area, which was home to 70.2% of Ontario’s immigrant population in 2016 (Statistics Canada 2019).

It has been shown in Canada that immigrants are typically healthier than those born in their host country, a phenomenon known as the “healthy immigrant effect”, although their health advantage tends to decline over the length of residency (Vang et al. 2017). The immigrant health advantage has been shown to extend to mortality (Ikram et al. 2016; D. W. R. Omariba 2015; Omariba et al. 2014b; Singh and Siahpush 2001). In Ontario specifically, immigrants experienced almost 60% lower all-cause mortality and premature mortality rates than their Canadian-born counterparts during 2002 to 2012 (Khan et al. 2017).

The health of immigrants is shaped by a combination of immigration policies and factors related to their country of origin and host country (Trovato 2017). Immigrants entering Canada are selected using a point system that favours applicants with high levels of educational attainment and language proficiency, in addition to health screening that covers communicable and chronic conditions (Kaushal and Lu 2015). In addition, new immigrants are likely to practice protective health behaviours that are shaped by cultural norms in their country of origin (Subedi and Rosenberg 2014; Vang et al. 2017). Studies have shown immigrant populations are less likely to have a chronic condition, smoke, be overweight or obese, or report their health as poor, relative to their native-born counterparts (De Maio 2010; Lu et al. 2017; Newbold and Neligan 2012; Wang and Hu 2013). The health trajectory of immigrants is influenced by a number of contextual factors, including access to and use of health services (Bissonnette et al. 2012; Harrington et al. 2013); the process of acculturation (Lara et al. 2005); and neighbourhood characteristics, such as the presence of people that share the same ethnicity (Bécares et al. 2009; Pickett and Wilkinson 2008).

The physical and social characteristics that are associated with neighbourhoods in which individuals live are known to affect health (Diez Roux and Mair 2010). In particular, areas with a high density of immigrants have demonstrated protective health effects in relation to lower obesity, lower cardiovascular disease-related hospitalizations, and better mental health outcomes (Emerson and Carbert 2017; Menezes et al. 2011; Omariba et al. 2014a). Furthermore, one study demonstrated that living in an immigrant neighbourhood, measured by the percent of immigrants in a census tract, was associated with lower mortality risk, which the authors described as a “healthy neighbourhood immigrant effect” (Ross et al. 2013).

When examining health variation at regional levels, the focus is often on health system factors that may be contributing to inequalities. It is currently not clear in the Ontario setting to what extent both the individual and contextual effects associated with immigrant density contributes to regional variations in mortality. This population-based study sought to describe the geographic variation in premature mortality in the province according to regional differences in immigrant populations to better understand these trends together.

## Methods

### Data sources

The study used population-based vital and demographic datasets that were linked using unique, encoded identifiers and analyzed at ICES. Death records were obtained from the Office of the Registrar General-Deaths (ORG-D) file, which covers all deaths that were registered in the province of Ontario from January 1, 1992, to December 31, 2012. The ORG-D was linked probabilistically to the Registered Persons Database (RPDB) with an overall linkage rate of 96.2% (Chiu et al. 2016). The RPDB is a central population registry file of all residents in Ontario who have ever received a health card number for the province’s universal health care system and contains basic demographic information, including sex, date of birth, and residential postal code. The Ontario portion of the Immigration, Refugees, and Citizenship Canada (IRCC) Permanent Resident Database was used to identify immigrants in our study population. The IRCC contains records for over 3 million individuals at the time of landing in Ontario from January 1985 to December 2012 and was linked to the RPDB with 86.4% overall linkage rate (Chiu et al. 2016). Information on immigrants arriving prior to 1985 is not captured in the IRCC Permanent Resident Database. In addition, the IRCC data available at ICES are not able to identify immigrants who landed in other provinces and moved to Ontario. Area-level measures of socio-economic status were calculated using data from the 2001, 2006, and 2011 Canadian census (Matheson et al. 2012). The study received ethics approval from the Research Ethics Boards at the University of Toronto and Sunnybrook Health Sciences Centre.

### Primary outcome

Our primary outcome was adult premature mortality, defined as all deaths between the ages of 18 and 74 years, from any cause. The upper age limit chosen for premature mortality is consistent with premature mortality indicator definitions applied in Canada (Canadian Institute for Health Information 2012).

### Population

The study population consisted of all adult premature deaths among Ontario residents, aged 18 to 74 years, for the period 1992 to 2012. Individuals were excluded from the cohort if their health card number was ineligible on their death date (*N* = 7006), if they were a non-Ontario resident (*N* = 1900), or if their age at death was less than 18 or greater than 74 years old (*N* = 1,009,938). After exclusions, the population-based cohort consisted of 674,028 registered deaths linked to the RPDB.

### Geographic regions

Using the last known postal code at time of death in the RPDB, individuals were assigned to a dissemination area (DA) and classified into geographic boundaries corresponding to Ontario’s 14 health service regions, known as Local Health Integration Networks (LHINs). We chose LHIN as the primary unit of analysis because it is the boundary that is used for health system planning evaluation in Ontario and we wanted our results to relate back to a planning level to demonstrate relevance of considering contextual factors at that level. We also present the population characteristics in five postal regions in the descriptive analysis according to the first letter of the postal code: Eastern (K), Greater Toronto and Hamilton (GTHA) Area (L), Toronto (M), Western (N), and Northern (P). These 5 levels were used to more feasibly describe the population across the Ontario geography. Supplement Figure 1 shows the relationship between geographic boundaries of the LHINs and postal regions.

### Covariates

#### Demographic and socio-economic factors

The study adjusted for year of death, age, and material deprivation quintile. Age at time of death was categorized as follows: 18–24, 25–34, 35–44, 45–54, 55–64, and 65–74 years old. Material deprivation score, obtained from the Ontario Marginalization Index (ON-Marg), is an area-level index that characterizes the ability of individuals and communities to obtain essential goods and services (Matheson et al. 2012). Specifically, the material deprivation dimension measures the proportion of the population within a geographic region that is low income, without high school diploma, lone-parent families, receiving government transfer payments, unemployed, and living in dwellings in need of repair. The 2001 and 2006 version of ON-Marg was developed using 2001 and 2006 Canadian census, respectively. In 2011, ON-Marg was based on alternative data sources in order to avoid data quality issues after the federal government replaced the mandatory census with a voluntary survey. Material deprivation scores were applied to each decedent according to their DA at time of death (2001 scores were applied to deaths that occurred between 1992 and 2003; 2006 scores for deaths between 2004 and 2008; and 2011 scores for deaths between 2009 and 2012). Individuals were subsequently categorized into material deprivation quintiles ranging from 1 (20% least deprived) to 5 (20% most deprived).

#### Immigration

Immigrant status was defined and measured at both the individual level and the aggregate level. At the individual level, immigrant status was defined as landed immigrants who were not Canadian citizens by birth and arrived in Ontario between 1985 and 2012. Immigrant status was a binary categorization. At the aggregate level, the proportion of immigrants in each LHIN region was operationalized as the *z*-score of the mean percentage of immigrants in Ontario in each year. This transformation indicates how many standard deviations an observation is above or below the mean. This standardized value was implemented to minimize directly interpreting the proportions, which has less generalizability, and instead describe their distance from the average level of immigrants in a region. Individuals were given an aggregate level z-score based on their place of residence at the time of their death. Numerators for the calculation of the percentage of immigrants living in the province were obtained from the RPDB linked to records in the IRCC’s Permanent Resident Database. Annual LHIN-specific population counts obtained from the RPDB were used as denominators. The IRCC database records indicated that from 1992 to 2012 the immigrant population in Ontario increased from 5.8% to 18.8%, with the largest proportion of immigrants residing within the LHIN regions located in Central Ontario (i.e., Central West, Central, Mississauga Halton, Toronto Central, and Central East) (see Table 1).

**Table 1:** Population characteristics and average annual premature mortality rate of Ontario’s Local Health Integration Network (LHIN) regions, 1992–2012

LHIN	Males			Females		
	Population estimate	Immigrants^a^, mean (%)	Average annual premature mortality rate	Population estimate	Immigrants^a^, mean (%)	Average annual premature mortality rate
[#] LHIN						
1. Erie St. Clair	232,470	6.3	5.31	229,517	6.3	3.47
2. South West	327,025	4.8	5.10	326,457	4.6	3.29
3. Waterloo Wellington	240,553	8.5	4.02	239,579	8.2	2.66
4. Hamilton Niagara Haldimand Brant	482,293	5.9	5.22	484,023	5.7	3.43
5. Central West	253,740	25.0	3.14	254,486	24.4	2.04
6. Mississauga Halton	361,351	21.6	3.03	371,077	21.3	2.04
7. Toronto Central	458,896	21.4	4.27	460,030	20.8	2.53
8. Central	543,754	24.5	3.14	568,129	24.6	2.04
9. Central East	520,531	17.4	4.11	535,513	17.5	2.73
10. South East	168,731	1.9	6.10	175,117	2.0	3.82
11. Champlain	426,670	7.5	4.18	438,589	7.5	2.76
12. North Simcoe Muskoka	140,032	2.2	5.52	140,785	2.3	3.45
13. North East	221,034	0.9	6.40	217,678	1.0	3.98
14. North West	93,175	1.4	5.67	90,019	1.6	3.62

*LHIN* Local Health Integration Network

^a^Average percentage of immigrants living in region in the period 1992–2012, obtained from Immigration, Refugees, and Citizenship Canada (IRCC) Permanent Resident Database

### Statistical analysis

We conducted separate analyses for females and males to account for sex-related differences in mortality observed in previous research (Rosella et al. 2016). The socio-demographic characteristics of adult individuals who experienced a premature death in Ontario between 1992 and 2012 were described using descriptive statistics.

We calculated the premature mortality rate per 1000 for cumulative years 1992 to 2012 in each postal region by using ORG-D death counts (numerator) and RPDB population counts in each year, for residents between 18 and 74 years old (denominator). We present the cumulative and average annual premature mortality rates by LHIN and the population characteristics by a larger area of geography.

To determine the contribution of immigrants to the variation in LHIN-level premature mortality between the geographic regions, we ran multilevel negative binomial regression analyses (Austin et al. 2018). These models treat LHIN as a random effect and we report the summary effect of the LHIN as a median rate ratio (MRR) instead of comparing each LHIN specifically. In our analysis, the MRR quantifies the heterogeneity in premature mortality rates between the 14 LHINs and was estimated from the variance of the LHIN-specific random effects. We first ran an unadjusted random effect model and then sequentially adjusted for age, material deprivation, and year, then added individual-level immigration and then area-level immigration status. We report the explained LHIN-level variation by calculating the percent change in the variance of the LHIN-specific random effects from the minimally adjusted model (i.e., age, year, material deprivation). The LHIN-specific events are also reported in Supplementary Table 1. Before running these models, the cohort was aggregated across the different categorical study covariates (i.e., year, material deprivation, age group, individual immigration status, and LHIN region). This created a dataset with 17,640 observations for the female dataset and 17,633 observations in the male dataset. The yearly standardized area-level immigrant proportion was linked by year and LHIN. The annual Ontario population aggregated across the different categorical study covariates divided by 1000 was used as the offset term, in order to account for the different population size across calendar years. Individuals (*N* = 4635 males, 4648 females) in the cohort with missing information on material deprivation or LHIN region were not included in the regression. We calculated rate ratios (RRs) and 95% confidence intervals (95% CIs) for the associations by taking the exponential of the beta coefficient from the multilevel regression model. All analyses were conducted using SAS statistical software, version 9.4, and regression models were run using the GLMMIX procedure.

## Results

### Descriptive statistics

The population sizes and immigration characteristics are shown in Table 1. Male average annual premature mortality rates ranged from 3.03 to 6.40 per 1000, and females ranged from 2.04 to 3.98 per 1000. Further socio-demographic characteristics of the cohort of premature deaths from 1992 to 2012 are described by five geographic regions in Ontario in Table 2. The largest proportions of immigrants resided in the Toronto (10.3%) and GTHA (5.3%) areas; these regions were also the most ethnically concentrated. In contrast, the lowest proportions of immigrants resided in the Northern (0.3%), Eastern (1.4%), and Western regions (1.8%). The age of premature death was slightly higher than the Ontario average in Eastern and Western Ontario. Relative to neighbourhoods with the highest material deprivation (Q5), the proportions of premature deaths were higher in Toronto and Northern regions compared with those of similar neighbourhoods in the GTHA, Western, and Eastern regions. The cumulative premature mortality rate per 1000 ranged from a low of 63.9 deaths in the Toronto area, and a high of 105.2 deaths in the Northern region.

**Table 2:** Cumulative premature mortality rate and distribution of characteristics in the cohort of premature deaths (< 75 years old), by Ontario geographic region, 1992–2012

Characteristic	Overall	K - Eastern	L - GTHA	M - Toronto	N - Western	P - Northern
*n*	674,028	117,963	201,203	135,341	149,528	69,993
Cumulative 20-year premature mortality rate (per 1000)	74.2	83.7	64.8	63.9	83.9	105.2
Average annual premature mortality rate (per 1000)	3.59	4.05	3.14	3.14	4.05	5.04
Sex						
Male	60.5	60.0	60.0	60.8	60.6	62.0
Female	39.5	40.0	40.0	39.2	39.4	38.0
Age (at death) (years, mean (SD))	60.7 (12.1)	61.2 (11.7)	60.4 (12.1)	60.4 (12.3)	60.9 (12.1)	60.5 (12.3)
18–24	1.6	1.4	1.6	1.3	1.7	2.1
25–34	3.0	2.5	2.9	3.5	2.9	3.1
35–44	6.5	5.8	6.7	7.4	6.1	6.0
45–54	14.1	13.6	14.9	14.1	13.6	13.7
55–64	26.2	26.6	26.6	25.3	26.1	26.6
65–74	48.6	50.1	47.3	48.3	49.6	48.5
Immigrant status						
Long-term resident	95.7	98.6	94.7	89.6	98.2	99.7
Immigrant < 5 years	1.0	0.3	1.1	2.4	0.4	0.1
Immigrant ≥ 5 years	3.4	1.1	4.2	7.9	1.4	0.2
Immigrant category*						
Economic class	28.4	27.9	29.6	28.0	25.8	36.3
Family class	50.7	47.5	53.4	50.2	44.5	47.1
Other	20.9	24.5	17.0	21.7	29.7	16.6
Material deprivation						
Q1 (least)	14.0	14.9	18.0	12.6	13.4	5.1
Q2	16.8	17.9	20.8	11.9	18.6	9.2
Q3	19.2	19.1	20.9	15.5	21.8	16.0
Q4	21.5	21.1	19.5	22.6	22.0	25.2
Q5 (most)	25.5	24.1	19.5	34.9	21.6	35.6
Missing	2.9	2.9	1.4	2.4	2.6	8.9
Ethnic concentration						
Q1 (least)	23.1	34.0	17.8	2.9	30.0	44.2
Q2	21.3	26.8	21.1	5.8	27.0	30.4
Q3	18.3	17.8	22.3	12.5	21.0	13.0
Q4	17.0	12.6	20.0	27.0	13.8	3.2
Q5 (most)	17.4	5.9	17.4	49.4	5.5	0.3
Missing	2.9	2.9	1.4	2.4	2.6	8.9

*Among individuals classified as immigrants only

### Regional variation in premature mortality in the multilevel model

The minimally adjusted negative binomial model confirmed regional variation in premature mortality in both sexes, after adjusting for year, age group, and material deprivation quintile in males (median RR 1.19, 95% CI 1.09–1.26) and in females (1.17, 95% CI 1.08–1.23), indicating significant LHIN variation persisted. These variations were attenuated after adjusting for individual and area-based immigration in males (RR 1.07, 95% CI 1.00–1.11) and in females (RR 1.04, 95% CI 1.00–1.05). These adjustments explained 84.1% and 94.4% of the LHIN-level variation in males and females respectively. Reduced risk for premature mortality was seen for immigrants compared with that for long-term residents in the fully adjusted models for both males 0.43 (95% CI 0.42, 0.44) and females 0.45 (0.44, 0.46) (Table 3, model 3). Additionally, one standard deviation increase from the provincial mean percentage of immigrants was associated with an 8% decrease in premature mortality among males (RR 0.92, 95% CI 0.89–0.94) and a 9% decrease among females (RR 0.91, 95% CI 0.89–0.93). The LHIN-specific results from the random effect model are available in Supplementary Table 1 and demonstrate similar attenuation across models.

**Table 3:** Median rate ratio (RR) of premature death in Ontario in multilevel count model, 1992–2012

	Model			
	Unadjusted RR (95% CI)	+ Age, material deprivation RR (95% CI)	+ Individual-level immigration RR (95% CI)	+ Immigrant- and area-level immigration RR (95% CI)
Males				
Median RR LHIN	1.14 (1.06, 1.19)	1.19 (1.09, 1.26)	1.11 (1.05, 1.15)	1.07 (1.00, 1.11)
Age				
18–24		0.03 (0.03, 0.03)	0.03 (0.03, 0.03)	0.03 (0.03, 0.03)
25–34		0.04 (0.03, 0.04)	0.03 (0.03, 0.03)	0.03 (0.03, 0.03)
35–44		0.06 (0.06, 0.06)	0.06 (0.06, 0.06)	0.06 (0.06, 0.06)
45–54		0.15 (0.15, 0.15)	0.15 (0.15, 0.15)	0.15 (0.15, 0.15)
55–64		0.39 (0.39, 0.40)	0.39 (0.38, 0.39)	0.39 (0.38, 0.39)
65–74, reference		1.00	1.00	1.00
Material deprivation				
1 (lowest)		0.61 (0.60, 0.63)	0.54 (0.53, 0.55)	0.55 (0.54, 0.55)
2		0.69 (0.68, 0.71)	0.62 (0.61, 0.64)	0.63 (0.62, 0.64)
3		0.76 (0.75, 0.78)	0.70 (0.69, 0.72)	0.70 (0.69, 0.72)
4		0.84 (0.83, 0.86)	0.80 (0.79, 0.82)	0.80 (0.79, 0.82)
5 (highest), reference Immigrant status vs. long-term residents Percentage of immigrants in area (*Z*-score)		1.00	1.00	1.00
			0.40 (0.39, 0.41)	0.43 (0.42, 0.44)
				0.92 (0.89, 0.94)
Explained LHIN-level variation (%)		Reference	65.5%	84.1%
Females				
Median RR LHIN	1.13 (1.05, 1.18)	1.17 (1.08, 1.23)	1.09 (1.04, 1.13)	1.04 (1.00, 1.05)
Age				
18–24		0.02 (0.02, 0.02)	0.02 (0.02, 0.02)	0.02 (0.02, 0.02)
25–34		0.03 (0.03, 0.03)	0.03 (0.03, 0.03)	0.03 (0.03, 0.03)
35–44		0.06 (0.06, 0.06)	0.06 (0.06, 0.06)	0.06 (0.06, 0.06)
45–54		0.16 (0.16, 0.16)	0.16 (0.15, 0.16)	0.16 (0.15, 0.16)
55–64		0.40 (0.39, 0.40)	0.39 (0.38, 0.40)	0.39 (0.38, 0.40)
65–74, reference		1.00	1.00	1.00
Material deprivation				
1 (lowest)		0.63 (0.62, 0.65)	0.58 (0.56, 0.59)	0.59 (0.58, 0.60)
2		0.70 (0.68, 0.71)	0.65 (0.63, 0.66)	0.65 (0.64, 0.66)
3		0.76 (0.74, 0.77)	0.72 (0.71, 0.73)	0.72 (0.71, 0.74)
4		0.83 (0.81, 0.84)	0.80 (0.78, 0.81)	0.80 (0.79, 0.82)
5 (highest), reference Immigrant status vs. long-term residents Percentage of immigrants in area (*Z*-score)		1.00	1.00	1.00
			0.42 (0.41, 0.43)	0.45 (0.44, 0.46)
				0.91 (0.89, 0.93)
Explained LHIN-level variation (%)		Reference	67.5	94.4

## Discussion

Using population-based linked mortality data, this study has demonstrated that the observed regional variation in premature mortality in Ontario, Canada, is patterned by regional differences in the immigrant population. Controlling for the effect of the area proportion of immigrants, LHIN-level variations in premature mortality persisted in both sexes, although the magnitude of premature mortality inequalities differed by sex. Our findings suggest a relationship between area-level contextual factors that co-exist in regions with large populations of immigrants and premature mortality.

Consistent with the literature on the immigrant health advantage, the findings corroborated reduced premature mortality rate among immigrants compared with long-term residents (Khan et al. 2017; D. W. R. Omariba 2015) and demonstrated a narrowing in the geographic differences in mortality after accounting for immigration. Immigrants to Ontario are primarily concentrated in the large census metropolitan cities located in Central Ontario; inherently, these urban areas have been shaped by immigrants in ways that support their health, such as the establishment of ethnic communities, access to same-language or culturally appropriate community supports, and cultural changes to the food environment (Qadeer et al. 2010; Rodriguez et al. 2016; Vézina and Houle 2017). These regions have also seen the greatest declines in premature mortality in Ontario (Buajitti et al. 2018). The protective health effects of neighbourhood immigrant concentration on the health of immigrants have been described in prior research, including healthy dietary patterns, reduced obesity, and better mental health outcomes (Alegría et al. 2017; Emerson and Carbert 2017; Park et al. 2011). One mechanism in which immigrant-populated areas may function to maintain the health profile of immigrants could be by the buffering of the acculturation process (Fox et al. 2017).

Our findings add to the evidence base emphasizing the importance of neighbourhood context and health (Diez Roux and Mair 2010). We demonstrate that the contextual effects associated with immigrant density on premature mortality are important factors in LHIN-level variation. Specifically, our results showed that regional-level increases in the immigrant population have lower rates of premature mortality. Previous research has observed that living in neighbourhoods with higher percentages of immigrants was associated with reduced rate of cardiovascular disease hospitalizations and all-cause mortality in both immigrants and non-immigrants, and in both sexes (Omariba et al. 2014a; Ross et al. 2013). Our results suggest that in the context of premature mortality, where deaths are largely preventable and associated with socio-economic and modifiable factors (Bauer et al. 2014; Stringhini et al. 2017), the physical and social attributes that exist in immigrant-populated regions may be sex-specific.

Our study is descriptive and does not elucidate how attributes that characterize immigrant dense areas collectively influence pathways that drive the observed geographic differences in premature mortality. The literature provides some insight into potential pathways worthy of further investigation in Ontario. One pathway noted is through local food environment which has been shown to vary by racial and ethnic composition (Moore and Diez Roux 2006). For example, one study from the United States found that neighbourhoods with higher immigrant composition had better access to healthy foods and were more associated with healthier dietary patterns compared with areas with a lower proportion of immigrants (Osypuk et al. 2009). The same study also found negative pathways to health in that immigrant neighbourhoods had lower walkability scores and were associated with lower levels of physical activity among Hispanics (Osypuk et al. 2009). The underlying role of employment and socio-economic mobility in immigrant- and non-immigrant-populated areas are also important considerations for untangling the reasons for regional variations in premature mortality (Mendez 2009).

Targets for reducing the global and regional premature mortality burden have been established as part of the United Nations Sustainable Goals for Health (Norheim et al. 2015). The findings of this study underscore that cross-jurisdictional comparisons of progress made towards reducing premature mortality need to be interpreted in the context of immigration policies. Our findings emphasize that measuring performance may be limited without considering the important effects of immigration. For example, it may be that immigration trends, rather than health system factors, are having a greater influence on health system outcomes.

A prominent strength of this study was the use of a large mortality database (ORG-D) that contained all deaths registered in Ontario over the study period, and the cohort was linked with an immigration population file (IRCC), which enabled accurate identification of those who immigrated into Canada after 1985. Our study population consisted of a large number of premature deaths of which a sizeable proportion were immigrants, allowing for a detailed assessment of premature mortality by immigrant status and Ontario region of residence. Our cohort originates from a Canadian province with the largest and most diverse population of immigrants, which may help contribute to the generalizability of the findings to other settings.

Our results should be interpreted with consideration of important study limitations. First, not all immigrants were identified in this analysis because the IRCC linkage starts in 1985 and does not include immigrants who initially landed in a province outside of Ontario. Our findings do not account for immigrants who could have landed in Ontario, but subsequently returned to their country of origin; if present, this could have artificially reduced the premature mortality rates observed among immigrants (Razum 2006). In our current analyses, we did not distinguish between immigration landing categories (e.g., economic, family, refugee class), which can be considered in future analyses to further our understanding of regional disparities in premature mortality. In addition, the geographic boundaries chosen for this analysis were defined to align with the broad health service planning areas that are frequently used for health system evaluation; however, they do not capture small-area differences in the immigrant population and can potentially mask spatial differences in premature mortality that exist between neighbourhoods. Finally, the ON-Marg 2011 uses different data sources than the ON-Marg 2001 and 2006, which could have potentially influenced material deprivation trends over time. However, given that neighbourhood characteristics change over time, we chose to reduce the potential for misclassification by using the 2011 ON-Marg to classify material deprivation in individuals who died in recent years of the study period (2009 to 2012).

## Conclusions

Immigration patterns are an important characteristic that should be considered when measuring population and health system outcomes. This is particularly true in Ontario, which has the largest population of immigrants in Canada, and where large proportions are concentrated in large cities that are centrally located. Our study demonstrated that the observed regional differences in premature mortality in Ontario are in part explained by individual-level effects associated with the health advantage of immigrants, as well as contextual area-level effects that are associated with regional differences in the immigrant population. Regional variation in premature mortality was attenuated after accounting for immigration and other factors; however, there are likely other important social and demographic factors that vary geographically and which could be contributing to the observed patterning.

## Electronic supplementary material

ESM 1 (PDF 748 kb)
