# Supplementary material for: Association between the regional variation in premature mortality and immigration in Ontario, Canada
Source: Can J Public Health. 2020 May 27;111(3):322–32. doi: 10.17269/s41997-020-00330-5 (PMC7351932; doi:10.17269/s41997-020-00330-5)
Supplement: Supplementary file 1 — (PDF 748 kb) [file 41997_2020_330_MOESM1_ESM.pdf]

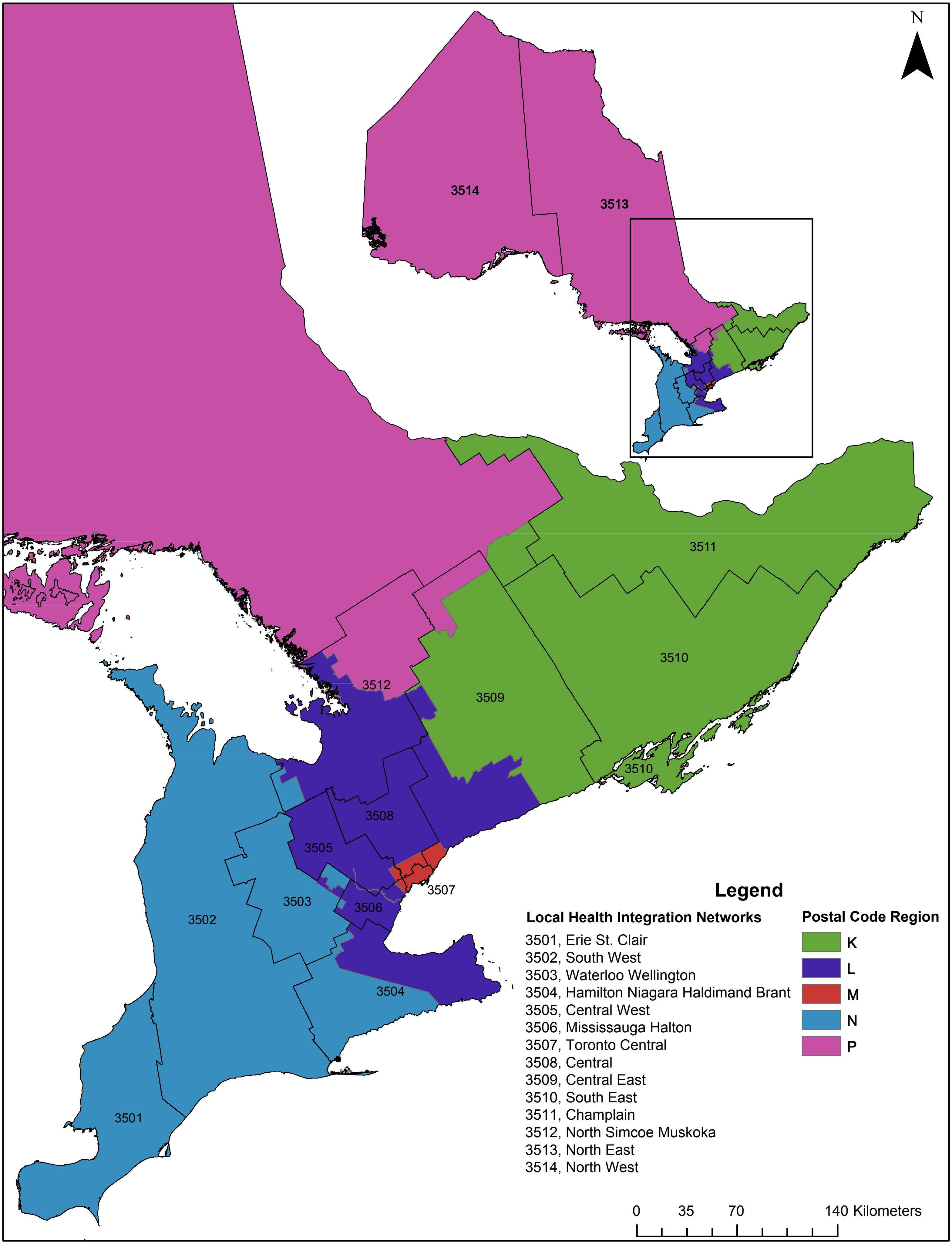
N
3514
3513
3511
3510
3512
3509
3510
3508
3505
3507
3503
3506
3502
3504
3501
Legend
Local Health Integration Networks
3501, Erie St. Clair
3502, South West
3503, Waterloo Wellington
3504, Hamilton Niagara Haldimand Brant
3505, Central West
3506, Mississauga Halton
3507, Toronto Central
3508, Central
3509, Central East
3510, South East
3511, Champlain
3512, North Simcoe Muskoka
3513, North East
3514, North West
Postal Code Region
K
L
M
N
P
0 35 70 140 Kilometers

Supplement Table 1. LHIN-level Random Effects

	Model			
	Unadjusted	+ Year, Age, Material Deprivation	+Individual Level Immigration	+ Individual and Area Level Immigration
**Males**	**RR (95% CI)**	**RR (95% CI)**	**RR (95% CI)**	**RR (95% CI)**
Central	0.79 (0.71 , 0.87)	0.70 (0.64 , 0.77)	0.81 (0.76 , 0.86)	0.90 (0.85 , 0.96)
Central East	0.91 (0.82 , 1.01)	0.85 (0.77 , 0.94)	0.94 (0.88 , 1.00)	0.99 (0.94 , 1.03)
Central West	0.87 (0.78 , 0.96)	0.80 (0.72 , 0.88)	0.88 (0.83 , 0.94)	0.99 (0.93 , 1.05)
Champlain	0.93 (0.85 , 1.03)	0.95 (0.86 , 1.05)	0.97 (0.91 , 1.03)	0.93 (0.89 , 0.97)
Erie St. Clair	1.09 (0.98 , 1.20)	1.14 (1.04 , 1.26)	1.12 (1.05 , 1.19)	1.06 (1.02 , 1.11)
Hamilton Niagara Haldimand Brant	1.03 (0.93 , 1.14)	1.05 (0.95 , 1.16)	1.06 (0.99 , 1.12)	1.00 (0.95 , 1.04)
Mississauga Halton	0.83 (0.75 , 0.92)	0.76 (0.69 , 0.84)	0.86 (0.81 , 0.91)	0.93 (0.88 , 0.98)
North East	1.22 (1.09 , 1.35)	1.25 (1.13 , 1.38)	1.13 (1.06 , 1.20)	1.02 (0.97 , 1.08)
North Simcoe Muskoka	1.12 (1.01 , 1.24)	1.17 (1.06 , 1.30)	1.09 (1.02 , 1.16)	1.00 (0.94 , 1.05)
North West	1.19 (1.07 , 1.32)	1.26 (1.14 , 1.39)	1.13 (1.06 , 1.20)	1.02 (0.97 , 1.08)
South East	1.14 (1.03 , 1.26)	1.21 (1.10 , 1.34)	1.12 (1.05 , 1.19)	1.02 (0.97 , 1.07)
South West	1.03 (0.94 , 1.14)	1.10 (1.00 , 1.22)	1.07 (1.01 , 1.14)	1.01 (0.96 , 1.05)
Toronto Central	0.98 (0.89 , 1.08)	0.98 (0.89 , 1.08)	1.08 (1.02 , 1.15)	1.19 (1.12 , 1.26)
Waterloo Wellington	0.98 (0.89 , 1.09)	1.00 (0.90 , 1.10)	1.00 (0.94 , 1.06)	0.97 (0.93 , 1.01)
**Females**				
Central	0.79 (0.72 , 0.88)	0.72 (0.66 , 0.79)	0.83 (0.79 , 0.88)	0.94 (0.90 , 0.98)
Central East	0.92 (0.84 , 1.02)	0.88 (0.80 , 0.96)	0.96 (0.91 , 1.02)	1.01 (0.98 , 1.05)
Central West	0.88 (0.80 , 0.98)	0.82 (0.75 , 0.89)	0.91 (0.86 , 0.96)	1.02 (0.98 , 1.06)
Champlain	0.95 (0.86 , 1.05)	0.99 (0.90 , 1.08)	1.00 (0.95 , 1.05)	0.96 (0.93 , 0.98)
Erie St. Clair	1.07 (0.96 , 1.18)	1.14 (1.04 , 1.24)	1.12 (1.06 , 1.18)	1.05 (1.02 , 1.08)
Hamilton Niagara Haldimand Brant	1.03 (0.93 , 1.14)	1.07 (0.98 , 1.17)	1.07 (1.01 , 1.13)	1.00 (0.97 , 1.03)

Mississauga Halton	0.85 (0.77 , 0.94)	0.81 (0.74 , 0.89)	0.91 (0.86 , 0.96)	0.99 (0.95 , 1.02)
North East	1.20 (1.08 , 1.34)	1.22 (1.11 , 1.33)	1.13 (1.07 , 1.19)	1.01 (0.98 , 1.05)
North Simcoe Muskoka	1.10 (0.99 , 1.23)	1.14 (1.04 , 1.24)	1.07 (1.01 , 1.13)	0.98 (0.94 , 1.01)
North West	1.21 (1.08 , 1.35)	1.22 (1.11 , 1.34)	1.13 (1.06 , 1.20)	1.02 (0.98 , 1.06)
South East	1.15 (1.03 , 1.28)	1.18 (1.08 , 1.29)	1.11 (1.05 , 1.17)	1.00 (0.97 , 1.04)
South West	1.03 (0.93 , 1.14)	1.10 (1.00 , 1.20)	1.07 (1.01 , 1.13)	0.99 (0.96 , 1.02)
Toronto Central	0.92 (0.83 , 1.01)	0.88 (0.81 , 0.96)	0.97 (0.92 , 1.03)	1.06 (1.03 , 1.10)
Waterloo Wellington	1.00 (0.90 , 1.10)	1.03 (0.94 , 1.13)	1.03 (0.97 , 1.09)	0.99 (0.96 , 1.02)
